# Degradation of Synthetic and Natural Textile Materials Using *Streptomyces* Strains: Model Compost and Genome Exploration for Potential Plastic-Degrading Enzymes

**DOI:** 10.3390/microorganisms13081800

**Published:** 2025-08-01

**Authors:** Vukašin Janković, Brana Pantelic, Marijana Ponjavic, Darka Marković, Maja Radetić, Jasmina Nikodinovic-Runic, Tatjana Ilic-Tomic

**Affiliations:** 1 Institute of Molecular Genetics and Genetic Engineering, University of Belgrade, Vojvode Stepe 444a, 11042 Belgrade, Serbia; vukasin.jankovic@imgge.bg.ac.rs (V.J.); brana.pantelic@imgge.bg.ac.rs (B.P.); marijana.ponjavic@imgge.bg.ac.rs (M.P.); jasmina.nikodinovic@imgge.bg.ac.rs (J.N.-R.); 2 Vinča Institute of Nuclear Sciences, National Institute of the Republic of Serbia, University of Belgrade, Mike Petrovića Alasa 12-14, Vinča, 11351 Belgrade, Serbia; darka.markovic@vin.bg.ac.rs; 3 Faculty of Technology and Metallurgy, University of Belgrade, Karnegijeva 4, 11000 Belgrade, Serbia; maja@tmf.bg.ac.rs

**Keywords:** bioremediation, compost, degradation, genome analysis, polyamide, *Streptomyces*, textile

## Abstract

Given the environmental significance of the textile industry, especially the accumulation of nondegradable materials, there is extensive development of greener approaches to fabric waste management. Here, we investigated the biodegradation potential of three *Streptomyces* strains in model compost on polyamide (PA) and polyamide-elastane (PA-EA) as synthetic, and on cotton (CO) as natural textile materials. Weight change of the materials was followed, while Fourier-Transform Infrared Spectroscopy (FTIR) and Scanning Electron Microscopy (SEM) were used to analyze surface changes of the materials upon biodegradation. The bioluminescence-based toxicity test employing *Aliivibrio fischeri* confirmed the ecological safety of the tested textiles. After 12 months, the increase of 10 and 16% weight loss, of PA-EA and PA, respectively, was observed in compost augmented with *Streptomyces* sp. BPS43. Additionally, a 14% increase in cotton degradation was recorded after 2 months in compost augmented with *Streptomyces* sp. NP10. Genome exploration of the strains was carried out for potential plastic-degrading enzymes. It highlighted BPS43 as the most versatile strain with specific amidases that show sequence identity to UMG-SP-1, UMG-SP-2, and UMG-SP-3 (polyurethane degrading enzymes identified from compost metagenome). Our results showcase the behavior of *Streptomyces* sp. BPS43 in the degradation of PA and PA-EA textiles in composting conditions, with enzymatic potential that could be further characterized and optimized for increased synthetic textile degradation.

## 1. Introduction

Nowadays, the textile industry ranks as the third most polluting sector globally, following petroleum and agriculture. In 2023, textile fiber production reached 124 million tonnes, marking a 7% rise from 2022, and is anticipated to hit 160 million tonnes by 2030 [1]. Synthetic fibers, mainly polyester, dominate production (57% of total fibers), while cotton constitutes 22% of total fiber [2]. And while virgin fiber production has increased, in general, pre- and post-consumer recycled textiles made up less than 1% of the worldwide material market in 2023 [2]. In addition, the textile industry also contributes significantly to plastic waste (42 million tonnes per year) and ocean pollution via microfibers [3]. Microplastics and microfibers predominantly originate from textile washing and substantially contribute to the contamination of soil and aquatic environments [4]. In addition, fast fashion has amplified environmental harm, reducing clothing durability and increasing waste production. Approximately 92 million tonnes of various textile waste are generated per year, according to the UNEP [5]. This is not surprising, considering that during a period of 15 years (2000–2015), clothing production doubled, but the duration of clothing use has dropped by 36% [5].

Polyamides (PA), known as nylons, are a type of synthetic fiber known for their high tensile strength, as well as being highly durable. PAs such as PA 6 (Nylon 6) or PA 6,6 (Nylon 6,6) are put to use in various fields such as textiles, entertainment, health, as well as in some more modern technological fields [6]. Currently, almost all PAs are destined for either landfill or incineration, because conventional methods of recycling do not meet economic incentives [7]. Polyurethane (PU) is a synthetic polymer class, used as starting materials in a range of industries such as packaging, furniture, textiles, as well as transportation, electronics, and adhesives [8]. Elastane (EA), the most common type of PU, is known for its exceptional elasticity. Elastane fibers are always mixed with one or more fibers in the technological process of knitting, for articles that require high elasticity [9]. Versatile and renewable, cotton (CO) is regarded as an extensively used natural material in textile, fashion, as well as in other applications [10]. PA, EA, and cotton fibers are usually coated, dyed, and mixed with various additives in order to increase the material properties, which makes existing recycling approaches ineffective.

The receptiveness of textiles to degradation mainly depends on their chemical structure. Low degradation of PA originates from its highly crystalline morphology [11]. Polymers, such as polyamides and polyurethanes, have a main chain that is not only C–C bonds but also contains atoms like O and N. With the existence of water, these polymers can be made more susceptible to degradation due to the presence of hydrophilic amide or ester bonds. So far, no enzyme has been characterized as an effective degrader of highly crystalline PA polymers [12]. Several enzymes reported to degrade low-molecular-weight polyamides (proteases, cutinases, and amidases) can only act on the surface of polyamides [13]. When it comes to microorganisms, Friedrich et al. detailed the degradation process of PA 6 with manganese peroxidase from the fungus *Bjerkandera adusta* [14]. On the other hand, bacterial degradation of PA 6 was reported by a thermophilic strain related to *Bacillus pallidus* [15]. Furthermore, the existence of several bacteria capable of using caprolactam from PA 6 production plant waste illustrates the degradation potential of bacteria in PA disposal [16].

PUs (such as EA) are mostly resilient to degradation by microorganisms because of their diverse chemical structures, made up of different isocyanates and polyols. However, studies of different fungal and bacterial degradation of PU have been reviewed [17]. PU was found to be prone to bacterial attack, and the degradation ability of *Bacillus subtilis* MZA-75 and *Pseudomonas aeruginosa* MZA-85 from soil was found to be limited to its soft segment in an individual capacity, with no evidence of an attack on the hard segment [8]. Very recently, a polyester PU-degrading *Bacillus* sp. YXP1 was identified. It was found that this bacterial strain is capable of PU depolymerisation, by hydrolysing ester and urethane bonds [18]. The degradation ability of PU was recorded for a couple of bacterial enzyme classes: ester hydrolases, nonpeptide amidases, lipases, ureases, and cutinases [19]. Also, PU-degrading lipases were identified from *Serratia* sp. HY-72, a strain isolated from an insect intestine [20]. On the other hand, being made of natural cellulose, CO materials are very susceptible to microorganism attacks, thus different methods of microbial and enzymatic degradation have been investigated in various soil [21] and composting environments [22].

Streptomycetes are a large group of mostly soil filamentous bacteria, characterized by a specific lifecycle, and they are spread across different environments of the world. Famous for their various antibiotic production and a wide range of bioactive compounds, they are also known to release enzymes that have numerous biotechnological applications [23,24]. In addition, they serve as a nonconventional host for recombinant protein production [25]. They have been described to biodegrade natural polymers such as chitin, lignin, and cellulose [26]. Regarding synthetic polymers, there have been studies that investigated the competence of *Streptomyces* as a degrader [27]. Additionally, an enzyme from a specific marine *Streptomyces* strain was found capable of hydrolyzing polyethylene terephthalate (PET), suggesting this research area is still undergoing early-stage development [28]. Recently, Verschoor et al. investigated the presence of bis(2-hydroxyethyl) terephthalate (BHET) degrading enzymes in *Streptomyces*, which can decompose (PET) and suggested a novel degrading enzyme family of *Streptomyces* [29].

In this work, we used the following textile fabrics: PA, PA-EA, and CO. Both synthetic (PA, PA-EA) and natural (CO) textile materials were subjected to a soil burial test augmented with *Streptomyces* spp. strains from an in-house collection, in order to study their biodegradation potential. This was achieved by determining material weight loss, while SEM and FTIR were used to analyze the morphology and the chemical structure of the materials, respectively. Ecotoxicological testing using the *Aliivibrio fischeri* bioluminescent biosensor, as well as cytotoxicity assessment using the healthy human cell line HaCaT, demonstrated that the textile extracts under investigation were environmentally nonhazardous and posed no ecological risk. By sequencing and analyzing bacterial genomes, a promising metabolic potential of three Streptomycetes was revealed, with a view to further characterizing and developing the efficient biocatalysts for increased textile degradation. This work addresses a biological approach to synthetic textile waste management, using *Streptomyces* bacteria in enhanced biodegradation composting conditions.

## 2. Materials and Methods

### 2.1. Chemicals

The components of the bacteriological media were of microbiological reagent-grade quality. All chemicals were of the quality of reagent-grade or higher and used without further purification. Solvents were utilized as received.

Mannitol, tryptic soy broth, tryptone, peptone, yeast extract, malt extract, and agar were purchased from Biolife Italiana (Milan, Italy). D-Glucose, NaCl, NH_4_Cl, C_6_H_10_FeNO_8_, Na_2_HPO_4_, KH_2_PO_4_, and carboxymethyl cellulose (CMC) were purchased from Fisher Scientific (Loughborough, UK). Dimethyl sulfoxide, N-Z Amine, CaCl_2,_ KCl, ZnSO_4_ × 7H_2_O, and lysozyme were obtained from Sigma Aldrich (Steinheim, Germany). K_2_HPO_4_ was obtained from Carlo Erba (Emmendingen, Germany). Acetone was purchased from Betahem (Belgrade, Serbia). PU polymer Impranil DLN^®^-SD (Impranil) was obtained from Covestro (Leverkusen, Germany). Soy flour was purchased from Bio Svet (Subotica, Serbia).

### 2.2. Textile Materials

Synthetic materials (knitted fabrics) comprised of PA 6,6 (Nylon 6,6) yarns (100%, 22 dtex, and 7 filaments), as well as EA yarns (44 dtex), covered with textured 22/7 PA 6,6 filaments (PA/EA, 1550 turns per meter), were both obtained from Fulgar East Ltd. (Zrenjanin, Serbia). Raw cotton (CO) fabric was purchased from the local wholesale supplier of textile fabrics with a weight of 128.98 g/m^2^.

### 2.3. Strain Selection and Characterization

From the in-house culture collection, three *Streptomyces* strains have been selected based on their cultivation ability on a variety of rich and minimal media, including *Streptomyces* sp. R1, previously mentioned as *Streptomyces* sp. RUJ1 [30], *Streptomyces* sp. BPS43, and *Streptomyces* sp. NP10 [31,32]. Suspensions of bacterial spores were made and maintained in 20% (*v*/*v*) glycerol, at −80 °C prior to the preparation of cultures [33].

Bacterial media used for general bacterial growth: mannitol soy flour (MSF) (20 g/L soy flour, 20 g/L mannitol, and 20 g/L agar); Luria–Bertani agar (LA) (10 g/L NaCl, 5 g/L yeast extract, 10 g/L tryptone, and 15 g/L agar); Sabouraud dextrose agar (SAB) (40 g/L glucose, 10 g/L peptone, and 20 g/L agar); and Tryptic soy broth (TSB) (20 g/L tryptic soy broth powder, and 20 g/L of agar). For the incubation of bacterial strains, as well as soil burial tests, a constant temperature of 30 °C was used.

For the purpose of screening bacterial strains, the following media were used: minimal salt medium (MSM) (1 g/L NH_4_Cl, 20 g/L of glucose, and 15 g/L agar. After autoclaving at 120 °C for 90 min, then 9 g/L Na_2_HPO_4_·12 H_2_O was added, as well as 1.5 g/L KH_2_PO_4_, 0.2 g/L CaCl_2_·2H_2_O, 0.2 g MgSO_4_·7 H_2_O, 0.0012 g/L Fe(III)NH_4_-citrate, 25% (*w*/*v*) N-Z amine–casein enzymatic hydrolysate prepared from bovine milk, and 0.1% (*v*/*v*) trace elements solution).

The cellulose and PU-degrading activity of three *Streptomyces* strains was tested prior to biodegradation studies by using a minimal solid medium enriched with substrates. Cellulase activity was followed by inoculating solid MSM containing 1% CMC as a cellulase substrate, and incubating it for 5 days at 30 °C [34]. For the staining of CMC, 0.1% Congo red solution was used. The plates were stained for 15 min, after which Congo red solution was poured, the plates were washed with distilled water, and then fixed with 1 M NaCl for 6 h at an ambient temperature. For the determination of PU-degrading ability of strains, the MSM medium was enriched with Impranil DLN^®^-SD dispersion (6 g/L), after which the strains were incubated at 30 °C for at least 2 weeks. The development of clearance in the form of a zone around the microorganisms was regarded as a positive result.

### 2.4. Textile Degradation in Model Compost

Textile materials used for the degradation study were cut into rectangular shapes, with dimensions of 5 cm × 3 cm. Samples were buried in model compost, following the previously adopted protocol [35]. Model compost was prepared from several different soil components: a field soil sample (from the Institute of Molecular Genetics and Genetic Engineering (Belgrade, Serbia)), two different commercially available compost mixtures (“Supstrat Maki” (Grgurevci, Serbia), and “Jablan” (Šabac, Serbia)). Dry soil samples were mixed evenly, and then water was added (soil to water ratio: 3:1). Model compost pH was determined by mixing 10 g of air dry soil with 10 mL of deionized water, and the mixture was centrifuged at 5000 rpms for 20 min, before the pH of the supernatant was measured using digital pH meter Hanna Instruments (Belgrade, Serbia). pH of the model compost before the incubation was approximately 5.8–5.9.

For the augmentation of compost, 20 mL of liquid precultures of *Streptomyces* strains were prepared in TSB, rinsed with phosphate buffer (pH 7), and then 1% inoculum was used to prepare 20 mL of bacteria cultures in TSB (incubated at 30 °C for 7 days), which were added periodically (15 days) to the augmented compost. This was also used to maintain the starting moisture of compost. For the nonaugmented compost, 20 mL of water was added instead of bacterial cultures.

Soil burial tests were done in plastic polypropylene containers (26 cm × 17 cm × 17 cm) stored at 30 °C in a thermal room. Containers were filled with a total of 1100 g of made model compost, with the samples being buried at a depth of 10 cm, as shown in the figure (Figure S1). The degradation of synthetic materials (PA and PA-EA) was followed over time: 4, 8, and 12 months, while the cotton degradation was kept for 1, 1.5, and 2 months.

Weight loss in the materials was measured at predefined time intervals. After the removal of the samples from the soil, they were washed with distilled water to remove the excess soil. Samples were dried at 37 °C until constant mass. Weight loss (%) was calculated: ((*m*_0_ − *m*_1_)/*m*_0_) × 100, where *m*_0_ represents the original mass of the tested material, and *m*_1_ refers to the mass of the material after a specific time interval. All the measurements were conducted in duplicates, and the final values were expressed as average values ± SD.

Microbial cell counts of the model compost were determined by dissolving 1 g of soil in 9 mL of phosphate buffer and making serial dilutions (10^−1^–10^−6^). Soil microorganisms were plated (100 µL) on three types of agar media: LA for heterotrophic bacteria, MSF for spore-forming bacteria, and SAB media for fungi. Dilutions with the countable number of colonies were counted after 24 h and expressed as colony-forming units per gram of soil (CFU/g). Results were presented in the Supplementary Material (Figures S4 and S5).

### 2.5. Characterization of Textile Materials

#### 2.5.1. The Fourier-Transform Infrared Spectroscopy (FTIR)

The Fourier-transform infrared spectroscopy (FTIR) was used to detect changes in the chemical structures of PA, PA-EA, and CO after the degradation. Dried samples were recorded using an IR-Affinity spectrophotometer (Thermo Fisher Scientific, NICOLET iS10, Waltham, MA, USA) in attenuated total reflection mode. The samples were analyzed in the wavenumber range of 4000 to 400 cm^−1^, at room temperature, with a resolution of 4 cm^−1^, using a fixed number of scans of 32.

#### 2.5.2. Field Emission Scanning Electron Microscopy (FESEM) Analysis

Materials morphology, before and after the degradation, was analyzed using FESEM. The images were captured using (FESEM, Mira3, Tescan, Brno, Czech Republic) with an accelerating voltage of 10 kV. Before the observation, samples were fixed on double-sided carbon adhesive tape on top of an aluminium stud and sputter-coated with a thin layer of gold.

### 2.6. Biological Evaluations

#### 2.6.1. *Aliivibrio fisheri* Toxicity Test

Aquatic toxicity was analyzed using BioFix^®^ Lumi Luminescent Bacteria kit (Macherey-Nagel GmbH & Co. KG, Duren, Germany), relying on bioluminescence inhibition of *Aliivibrio fischeri* (also known as *Vibrio fischeri*) based on the ISO 11348-3 standard. In brief, PA, PA-EA, and CO material extracts were prepared by immersing sample threads (the samples were aseptically disintegrated into threads) in the provided BioFix Lumi Medium (10 mg/mL) and incubating at 37 °C for 72 h and 180 rpm. Suspensions were centrifuged for 10 min at 5000 rpm (Eppendorf Centrifuge 5804R, Eppendorf, Hamburg, Germany), and the filtered supernatants were used as material extracts. Prior to testing, freeze-dried *A. fischeri* were reconstituted in BioFix Lumi Medium and incubated for 30 min at 15 °C. The material extracts were then mixed with the bacterial suspension in a 1:1 ratio and incubated at 15 °C. The change in bioluminescence was measured after 30 min, and toxicity was determined by calculating the percentage of bioluminescence inhibition. All tests were performed in triplicate, and the extent of bioluminescence inhibition was measured using a GloMax^®^ 20/20 Luminometer (Promega Corporation, Madison, WI, USA).

#### 2.6.2. Cytotoxicity Assay

The cytotoxicity of the PA, PA-EA, and CO was tested on human keratinocyte cells (HaCaT cell line obtained from ATCC), in accordance with a method described previously [36]. The MTT reduction assay (3-(4,5-dimethylthiazol- 2-yl)-2,5-diphenyltetrazoliumbromide- MTT) was performed in quadruplicate, and results were expressed as percentages relative to the control (untreated cells), which were arbitrarily set to 100%. The cell viability rate (%) was calculated: (OD of the treated group/OD of the control group) × 100. Results are presented as means ± SD.

GraphPad Prism 9.4.1 software (GraphPad Prism 9.4.1, La Jolla, CA, USA) was used to perform a one-way analysis of variance (ANOVA), followed by Tukey’s multiple comparisons test. The results are presented as mean ± standard deviations (SD). The difference was statistically significant at *p* ≤ 0.05.

### 2.7. Degradation Products Analysis Using Liquid Chromatography—Mass Spectrometry

Strain *Streptomyces* sp. BPS43 was grown in MSM medium supplemented with glucose (20 g/L) at 30 °C, 180 rpm for 7 days. Preparations of the cell-free extract (CFE) and supernatant were done following the procedure of [37]. Pieces of PA, PA-EA material (1 cm × 1 cm) were immersed in the mixture of supernatant and CFE in a total volume of 5 mL (ratio as in the culture conditions) and incubated at a speed of 180 rpm, at 30 °C for 5 days. Soluble degradation products were identified using a TSQ Fortis TM Plus triple quadrupole mass spectrometer coupled with a Vanquish™ Core HPLC System (Thermo Fisher Scientific, Waltham, MA, USA). Separation occurred on an Acclaim™ Polar Advantage II C18 column. The method, based on Mustuga et al. [38] with minor changes, used a mobile phase of water and acetonitrile (both with 1% formic acid). Separation was performed at 0.3 µL/min with a gradient starting at 99% water. Detection was carried out in single ion monitoring (using masses of known PA and PU degradation products displayed in Table S1) [38,39] with positive spray voltage at 3500 V. Data was analyzed using Xcalibur 3.1, subtracting control spectra and comparing the masses to hypothetical degradation products.

### 2.8. Streptomyces Genome Sequencing and Analysis

Complete genomes of strains *Strepomyces* sp. R1, *Streptomyces* sp. BPS43 and *Streptomyces* sp. NP10 was sequenced by Novogene GmbH (Munich, Germany) using the Illumina sequencing platform. Adapters and low-quality nucleotides (quality score < 30) were removed by Cutadapt v. 4.9 [40], and de novo assembled using SPAdes v. 3.15.3 (St. Petersburg genome assembler) [41]. The assessment of genome assemblies was conducted with BUSCO (Benchmarking Universal Single-Copy Orthologs) [42] using the Streptomycetales lineage and QUAST (Quality Assessment Tool for Genome Assemblies) [43]. The annotation and functional analysis of selected genomes was conducted using the Joint Genomes Institute (JGI) pipeline v. 5.0.0. The genomes were deposited at JGI with accession numbers: 8122401480 and 8122886195, while *Streptomyces* sp. NP10 was sequenced previously [31] and deposited at GenBank (accession number: PDIQ01). For KEGG (Kyoto Encyclopedia of Genes and Genomes) pathway and additional functional annotation and protein-coding genes, blastKOALA v. 3.1 (KEGG Orthology And Links Annotation) [44] was used.

Homologous enzymes capable of polymer degradation were picked out by blasting (query coverage > 80% and >40% identity) against the PAZy (The Plastics-Active Enzyme Database) [45] and PlasticsDB (Plastic Biodegradation Database) [46]. Cellulases were identified using dbCAN3. The initial hits from the Carbohydrate-active database (CAZy) were filtered for those with a three-tool consensus (HMMER, dbCAN subfamily, and DIAMOND), an adequate EC number (EC 3.2.1.4), and a signal peptide. For the assembly of the phylogenetic tree, MEGA 12 (Molecular Evolutionary Genetic Analysis) software was used.

## 3. Results

### 3.1. Characterization of Selected Streptomyces Strains

**Figure 1:**
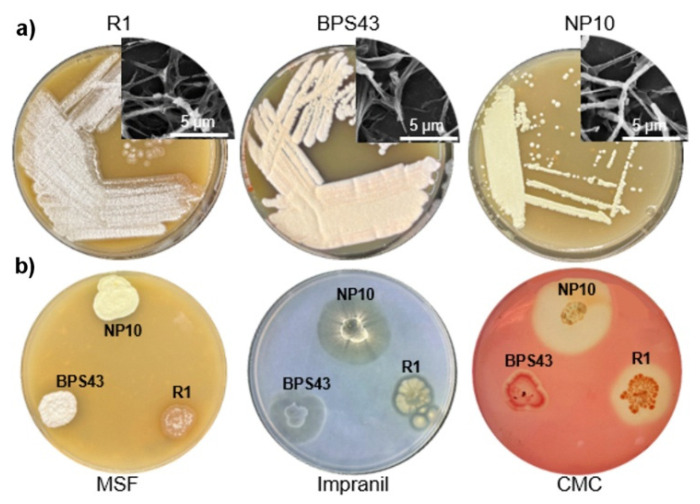
The appearance of *Streptomyces* sp. R1, *Streptomyces* sp. BPS43, and *Streptomyces* sp. NP10. (**a**) Incubated on MSF plates with FESEM images of *Streptomyces* sp. R1, *Streptomyces* sp. BPS43 and *Streptomyces* sp. NP10, respectively (at a magnification of 13,000×). (**b**) Incubated on MSF plates, MSM medium plates with Impranil DLN^®^-SD, and MSM medium plates with CMC plates.

After strain characterization, isolation, and screening of the in-house *Streptomyces* collection [30], three strains were selected for this study, namely *Streptomyces* sp. R1, *Streptomyces* sp. BPS43 and *Streptomyces* sp. NP10 (Figure 1). *Streptomyces* sp. R1 was isolated from the bark of the smoke tree, *Cotinus coggygria* [30]. *Streptomyces* sp. BPS43 was obtained from the samples of soil collected from a former vineyard in Serbia. Finally, *Streptomyces* sp. NP10 was isolated from a soil underneath a decaying wood in a village of Čumić, Serbia, which is previously associated with the production of long-chain fatty acids [31]. These strains could grow and sporulate abundantly on standard complex media containing soy flour within 5 days at 30 °C, and their spores were soft white-yellow for *Streptomyces* sp. NP10; light pink with a rough texture for *Streptomyces* sp. R1; and dark pink spores with smooth surfaces for *Streptomyces* sp. BPS43 (Figure 1a). All showed characteristic branched mycelia for *Streptomyces* spp. determined by SEM. All three were tested to see if they could depolymerize impranil DLN^®^-SD, a model substrate for polyurethanes. *Streptomyces* sp. BPS43 showed the largest zones of clearance when Impranil was used as a substrate (radius of 8.75 ± 1.25 mm), while *Streptomyces* sp. NP10 showed the highest activity on the CMC substrate (radius of 11.75 ± 0.65 mm) (Figure 1b).

### 3.2. Textile Degradation in Compost

**Figure 2:**
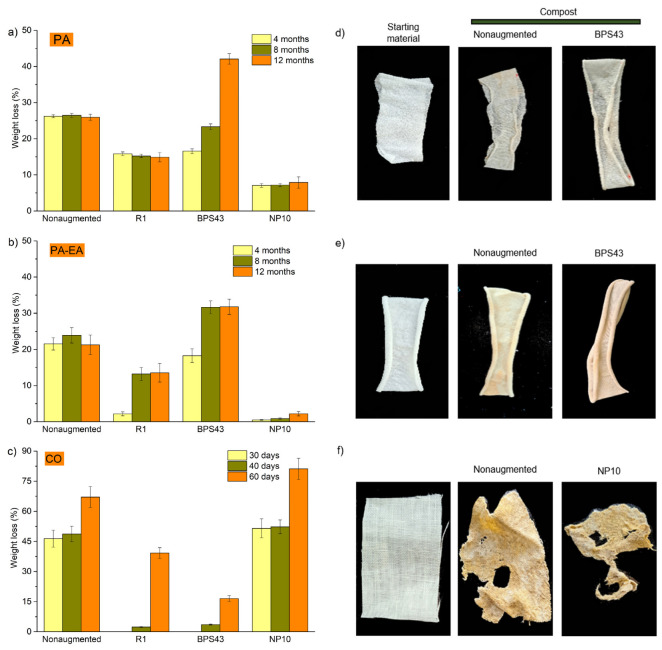
Weight loss (%) of textile samples upon incubation in bioaugmented model compost: (**a**) PA, (**b**) PA-EA, and (**c**) CO. Macroscopic appearance of textile samples before and after burial: (**d**) PA materials buried for 12 months (nonaugmented; BPS43—compost augmented with *Streptomyces* sp. BPS43); (**e**) PA-EA samples buried for 12 months (nonaugmented; BPS43—compost augmented with *Streptomyces* sp. BPS43); (**f**) CO samples buried for 30 days (nonaugmented; NP10—compost augmented with *Streptomyces* sp. NP10).

The weights of textile samples degraded in model compost, both nonaugmented and augmented with *Streptomyces* sp. R1, *Streptomyces* sp. BPS43, and *Streptomyces* sp. NP10, were assessed at predetermined time intervals. Weight loss values for PA, PA-EA, and CO materials, degraded in nonaugmented and augmented model compost, are presented in Figure 2. The sample’s appearance, before and after degradation, as well as its weight loss upon incubation in augmented compost, compared to the nonaugmented compost, can be observed in Figure 2. Upon incubation in model compost containing unidentified microorganisms, the degradation of synthetic materials occurred within the first 4 months of up to approximately 26% weight loss detected for PA, with no further progression in degradation with prolonged time, while weight loss in PA-EA was the highest after 8 months (~23% weight loss) (Figure 2b). Bioaugmentation with *Streptomyces* sp. BPS43 resulted in lower PA weight loss after 4 months (9%) compared to degradation in nonaugmented compost, revealing that supplementation by BPS43 did not accelerate the degradation for this time interval. However, after 12 months of degradation, in the presence of *Streptomyces* sp. BPS43, PA samples indicated remarkably higher weight loss (16%) compared to nonaugmented compost, resulting in an overall PA weight loss of 42% (Figure 2a).

The augmentation by *Streptomyces* sp. BPS43 also improved the degradation of PA-EA textile after 8 and 12 months, resulting in a 7% and 10% increase in weight loss, respectively (Figure 2b). Overall, a PA-EA weight loss of 31% was obtained. On the contrary, *Streptomyces* sp. R1 and *Streptomyces* sp.NP10 bioaugmentation resulted in lower weight loss compared to the nonaugmented model compost, with the biodegradation of PA (*Streptomyces* sp. R1) of ~11% and PA-EA (*Streptomyces* sp. NP10) of ~19%, after 12 months (Figure 2a,b). This might suggest the possible competition between indigenous microbes of the model compost with the selected two strains (*Streptomyces* sp. R1 and *Streptomyces* sp. NP10) for growth resources. For the accelerated degradation in model compost, *Streptomyces* sp. BPS43 strain could be suggested as a promising augmentation agent.

After the degradation, the macroscopic appearance of PA materials showed negligible color changes, both in nonaugmented and augmented compost (Figure 2d and Figure S2). For PA-EA, visible change in coloration (from white to yellow) was evident both in the case of samples degraded in augmented and nonaugmented compost (Figure 2e and Figure S2).

When CO textile was treated under the same conditions applied for PA and PA-EA, but for a shorter time of degradation, the overall weight loss, in nonaugmented compost, increased from 46% weight loss within 1 month to 67% after the additional month (Figure 2c). The augmentation with *Streptomyces* sp. NP10 enhanced the degradation, resulting in a higher weight loss of 14%, measured after 2 months (Figure 2c). The addition of *Streptomyces* sp. R1 and *Streptomyces* sp. BPS43 resulted in 27 and 50% lower degradation of cotton, respectively, in comparison to nonaugmented soil (Figure 2c).

After 60 days of burial in compost augmented with *Streptomyces* sp. NP10, visualization of the samples revealed almost complete disintegration of the CO material, with smaller fragments left (Figure 2f). However, even after 30 days of degradation in nonaugmented, as well as augmented model compost conditions, the changes in the sample’s appearance were visible (Figure S3).

A change in pH value of model compost was recorded. After 12 months of the soil burial test, the pH value of model compost has increased from 5.8–5.9 to around 7, both for noaugmented, and model compost augmented with *Streptomyces* sp. BPS43.

The total number of cultivable microorganisms was counted for nonaugmented model compost (before and after the burial test) and model compost augmented with *Streptomyces* sp. BPS43 (Figures S4 and S5). From the graph, it could be seen that after 12 months, there is no significant change in the number of microorganisms for the nonaugmented model compost, but there is an increase in the number of cultivable microorganisms in model compost augmented with *Streptomyces* sp. BPS43 (Figure S4).

### 3.3. Textile Surface Analysis

**Figure 3:**
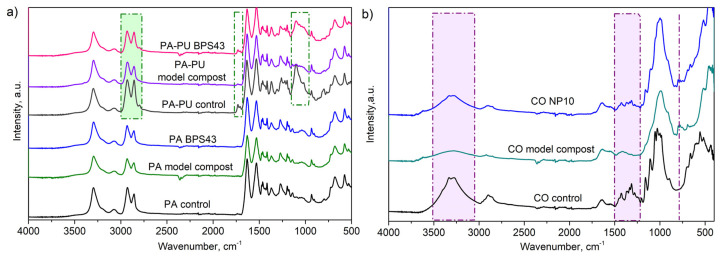
FTIR spectra of textile materials acquired after burial tests: (**a**) 12 months of incubation for PA and PA-EA materials; (**b**) 60 days of incubation for CO materials.

Changes in the chemical structure of degraded textile materials were assessed by FTIR, and the representative spectra are presented in Figure 3. In the FTIR spectrum of PA starting (control) sample, characteristic absorption peaks representative of the polyamide structure were detected (Figure 3a). From the presented FTIR spectra of PA material degraded in augmented compost (with *Streptomyces* sp. BPS43), the structure of the polymer remained unchanged. This could be attributed to the small weight loss in the material (16% after 12 months compared to the nonaugmented compost), and these small changes were probably below the sensitivity limit to be detected. In the case of the FTIR spectrum of a PA-EA control textile material, all PA characteristic peaks were detected; most of them overlapped with the EA characteristic bands, and the only registered additional peak came from the urethane group at 1730 cm^−1^. The relative intensities and positions of the peaks depend on the composition of the blend. Analyzing the spectra of PA-EA material degraded in augmented model compost, the intensity of the characteristic peaks ranging from 3000 to 2800 cm^−1^ has decreased (Figure 3a). A decrease in the peak intensity located at 1097 cm^−1^ was also detected (Figure 3a). These results showed that the PA-EA sample degraded in augmented compost, which expressed weight loss (7 wt% after 8 months, and 10 wt% after 12 months, compared to the nonaugmented compost), similar to a weight loss in PA, also exhibited chemical changes in the material structure.

The remarkable disintegration of CO samples (Figure 2c,f) is also reflected in their structure, which was confirmed by the decrease and absence of characteristic peaks in FTIR (Figure 3b). Analyzing the FTIR spectrum of the material after degradation, there is an evident decrease of the characteristic peaks at 3308 cm^−1^ and 2892 cm^−1^. Additionally, the most intensive changes were observed for the bands at 1160 and 1100 cm^−1^, indicating that ether bonds are quite susceptible to degradation and breakage between glucose units (Figure 3b). All aforementioned results suggested that CO textile material, a natural biopolymer, had better degradation behavior in comparison to the PA and PA-EA synthetic materials.

**Figure 4:**
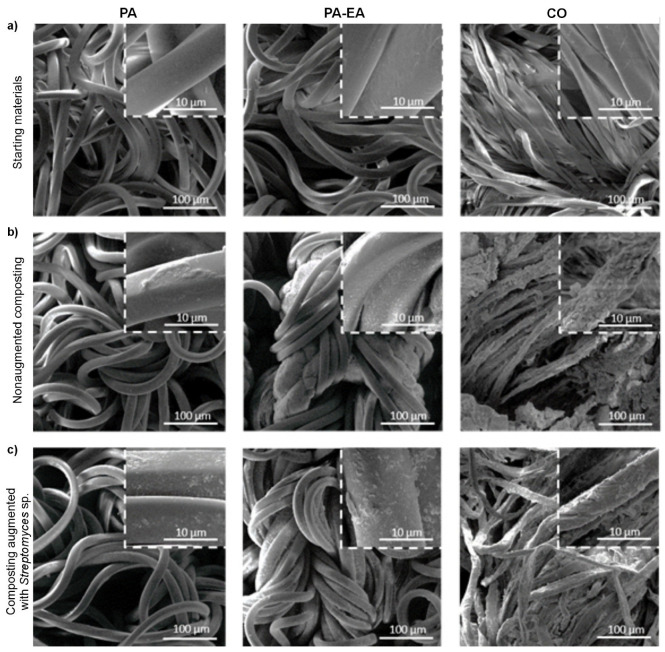
FESEM images of: PA, PA-EA, and CO textile samples (**a**) before burial; (**b**) in nonaugmented model compost; (**c**) in augmented model compost (at a magnification of 500× with the corresponding inserted images at a magnification of 5000×).

The morphology of textiles before and after degradation in nonaugmented and augmented model compost was observed by FESEM (Figure 4). The representative microphotographies of control samples showed the characteristic morphology of PA, PA-EA, and CO material before degradation, respectively (Figure 4a). All buried samples showed visible morphological changes post-degradation (Figure 4b,c). For PA material degraded in the augmented compost, the fibers appeared rougher, whereas the PA from nonaugmented compost indicated less prominent but detectable changes in the surface morphology. In the case of PA-EA textile material, more progressive changes in morphology were noticed. Eroded fibers with rough morphology and irregularities were observable, especially upon incubation in augmented compost at higher magnification (Figure 4, inlets). CO fibers typically have a relatively smooth surface, but after the burial of cotton material in nonaugmented model compost, pronounced changes in the fiber morphology were visible due to the substantial degradation under the tested conditions (Figure 4b). Although samples were subjected to washing after the degradation experiments, the compost residues remained visible due to the high adhesion to CO.

### 3.4. Eco- and Cytotoxicity of the Textile Material Extracts

**Figure 5:**
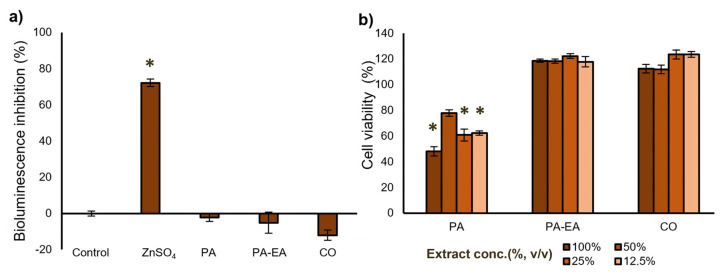
(**a**) Inhibition of *A. fischeri* bioluminescence upon exposure to PA, PA-EA, and CO extracts (average ± the SD, and the comparison to the untreated control and a reference compound ZnSO_4_ × 7H_2_O). (**b**) Cytotoxicity of PA, PA-EA, and CO extracts towards HaCaT cell line (* = *p* ≤ 0.05).

To investigate the ecotoxicity of the textile material extracts obtained from PA, PA-EA, and CO, the bioassay based on the bioluminescence inhibition of the marine bacterium *A. fischeri* was carried out. Toxic compounds cause the inhibition of the luciferase enzyme system, which is manifested in the rapid reduction of bioluminescence emission of the bacterium. Reduction aligns with toxicity level, enabling clear measurement. This reduction is related to the potency of the toxicant, providing a standardized evaluation outcome. Results in Figure 5a showed evidence of the complete absence of ecotoxicity of 100% material extracts PA, PA-EA, and CO, compared to reference compound zinc sulfate heptahydrate (ZnSO_4_ × 7H_2_O). Zinc sulfate heptahydrate’s ability to inhibit growth is also used to validate the test, and the obtained results were comparable to previously published data (ISO 11348-3). Material extracts PA, PA-EA, and CO did not show any toxic effect on *A. fischeri*, indicating that there was no undesirable influence on the bacterial bioluminescence. The results of BioFix^®^ Lumi tests clearly showed that tested material extracts are environmentally nonthreatening and ecologically harmless.

To confirm the nontoxic nature of PA, PA-EA, and CO material extracts, a cytotoxicity test was also employed on healthy human keratinocytes (HaCaT) as a predominant cell type in the epidermis of the skin. As evident from Figure 5b, the highest concentration of PA extract exhibited a moderate cytotoxic effect on HaCaT cells and resulted in approximately 50% cell survival, whereas a 50% dilution reduced the HaCaT cell survival to 77%. Interestingly, PA-EA and CO extracts at all tested concentrations promoted cell viability exceeding 100%, indicating noncytotoxicity under the tested conditions. Based on the ISO 10993-5 standard, cell viability exceeding 80% indicates noncytotoxicity.

### 3.5. LC-MS Analysis of Degradation Products

To further investigate the degradation of synthetic fibers by *Streptomyces* sp. BPS43, reactions with CFE were set up, and the reaction mixture was analyzed using LC-MS. The PA6 dimer (227.3 *m*/*z*) was found in both controls and reaction samples, indicating it leaches directly from the materials. The most significant change was the increased detection of the PA6 tetramer (453.3 *m*/*z*) in the reaction mixtures, as shown in Figure S6. This was observed in both PA and PA-EA samples, suggesting the changes seen in the fibers are primarily from PA degradation, and the PU segment remains intact, as no known PU degradation products were detected. However, due to the complex structure of PUs, other degradation products might have been released; their identification and detection are outside the scope of this study.

### 3.6. Genomic Exploration of Three Streptomyces

**Figure 6:**
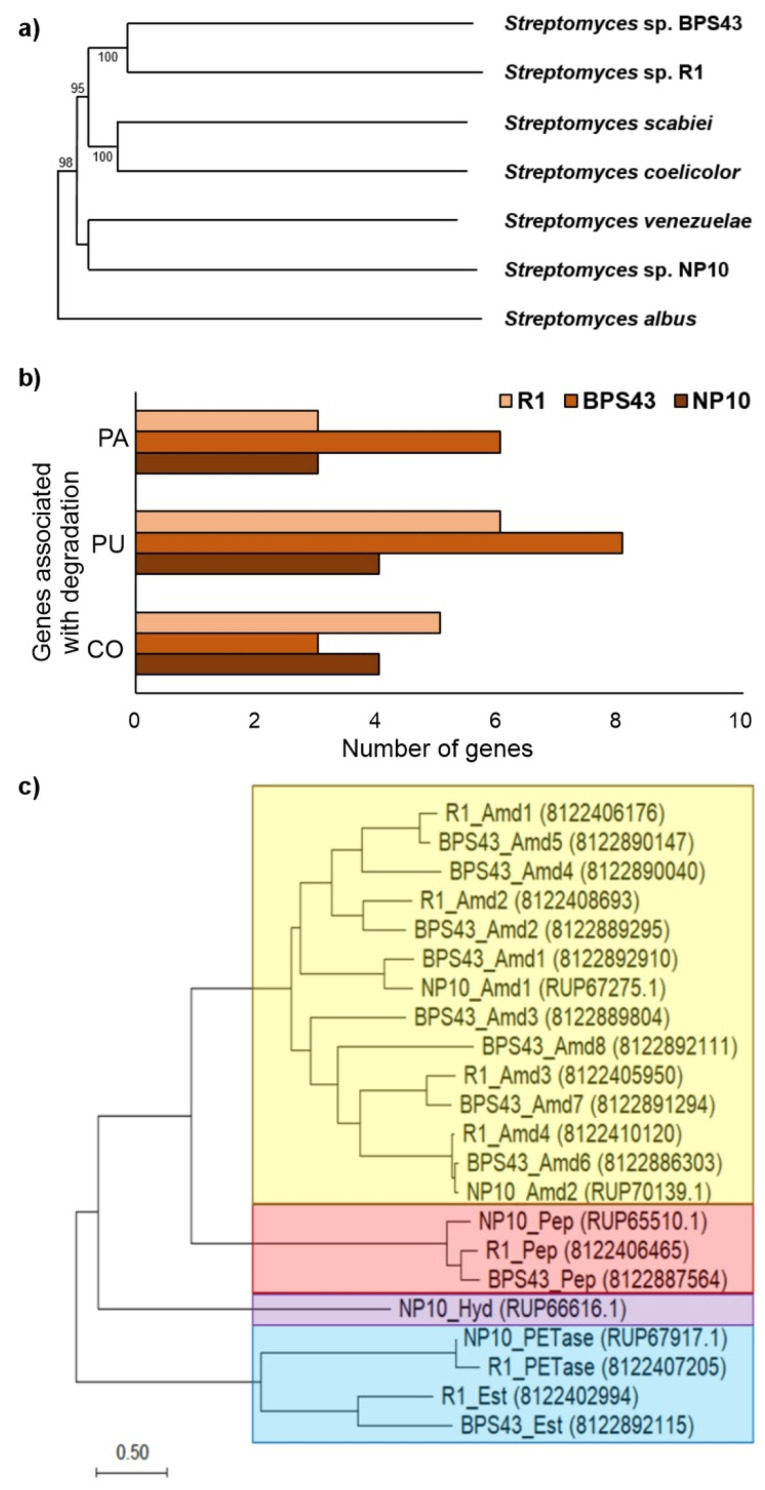
(**a**) Phylogenetic tree showcasing the evolutionary relationship between *Streptomyces* sp. R1, *S. spectabilis* BPS43, *S. rubiginosohelvolus* NP10, and reference *Streptomyces* spp. (**b**) The total number of PA, PU, and CO degrading enzymes found in three *Streptomyces* genomes. (**c**) Phylogenetic tree of PA-degrading and PU-degrading enzymes extracted from *Streptomyces* sp. R1, *S. spectabilis* BPS43 and *S. rubiginosohelvolus* NP10 (presented as enzyme class based on InterProScan predictions: yellow area (Amidases-Amd); red area (Peptidases-Pep); purple area (Hydrolases-Hyd); and blue area (PETases and Esterases-Est)).

Whole genome sequencing was performed to elucidate the observed biodegradation potential of selected *Streptomyces* strains and to further study their biocatalytic potential. The genomes ranged between 7.7 and 9.9 Mbp, 70.2–72.4% GC content, and were >99% complete according to BUSCO, indicating high-quality assemblies. The enzymatic potential of the strains was displayed between 6850 and 9120 protein-coding genes. An overview of the genome characteristics is presented in Table S2. The strains were taxonomically identified by multi-locus sequence typing (MLST) [47] as *Streptomyces rubiginosohelvolus* for NP10, *Streptomyces spectabilis* for BPS43, while *Streptomyces* sp. R1 seems to be a new species with the highest similarity to *Streptomyces rectiviolaceus* and *Streptomyces durmitorensis* (42%). A phylogenetic tree based on the whole genomes showcased the evolutionary relationship between these strains and some of the best-studied *Streptomyces* strains as references: *S. coelicolor* A3, *S. scabiei* RL-34, *S. venezuelae* ATCC 10712, and *S. albus* DSM 40,763 [48]. R1 and BPS43 clustered together were the closest to *S. coelicolor* and *S. scabiaei*, while NP10 was the closest to *S. venezuelae* (Figure 6a).

Based on KEGG pathway annotations, no discernible difference between the three strains could be observed (Figure S7); thus, the strains were further analyzed in terms of enzymes relevant for synthetic and natural textile degradation. An abundance of putative hydrolases was detected in the three genomes, with 458 in *Streptomyces* sp. R1, 414 in *S. spectabilis* BPS43, and 688 in *S. rubiginosohelvolus* NP10 (Table S3). Of these, between 13 and 22% had the ability to act on C-N bonds. This corresponds to 102 for *Streptomyces* sp. R1, 87 for *S. spectabilis* BPS43, and 94 for *S. rubiginosohelvolus* NP10. To further narrow down the enzymes responsible for the degrading activity, the proteomes of the three strains were compared to known PA/PU degrading enzymes, PU being the large class of polymers of which EA is a type and used in this study. A detailed list of PA/PU degrading homologs is displayed in Table S4. The analysis revealed multiple putative PA/PU-degrading enzymes with a total of 22 homologs, out of which *S. spectabilis* BPS43 had the highest number: 14 (six for PA and eight for PU), followed by *Streptomyces* sp. R1 with 9 (three for PA and six for PU), while, despite having the most hydrolases, *S. rubiginosohelvolus* NP10 had only seven PA/PU degrading homologs (three for PA and four for PU) (Figure 6b).

The investigation of the evolutionary relationship of PA/PU-degrading homologs in the designated strains and the identification of strain-specific enzymes was achieved with a maximum likelihood phylogenetic tree. The sequences were distinctly grouped into four primary branches, corresponding to amidases, peptidases, hydrolases, and esterases (Figure 6c).

Analyzing the genomes of the *Streptomyces* strains, we identified between 350 and 500 carbohydrate enzyme-related genes involved in carbohydrate metabolism and degradation (Table S5). The most abundant are glycoside hydrolases (GHs). Out of all the GH, 13 were identified as cellulases (EC 3.2.1.4): six for *Streptomyces* sp. R1, three for *S. spectabilis* BPS43, and four for *S. rubiginosohelvolus* NP10 (Figure 6b). Among these, the most abundant family of enzymes involved in cellulose breakdown was the glycoside hydrolase family 6 (GH6); however, members of the GH5 family have been identified as well (Table S5). GH6 family members cleave β-1,4 glycosidic bonds in cellulose/β-1,4-glucans [49]. Out of 14 total GH6 family members identified, 5 were found in *Streptomyces* sp. R1, 3 were found in *S. spectabilis* BPS43, and 4 were found in *S. rubiginosohelvolus* NP10. As for noncatalytic modules, several different carbohydrate-binding modules have been discovered (CBM2, CBM3, CBM5, CBM6, CBM10, and CBM16). Of these, CBM2 is the largest in prokaryotes and contains members that bind cellulose, xylan, as well as chitin [49,50]. Additionally, CBM2 is the most dominant among selected strains, with five in *Streptomyces* sp. R1, two in *S. spectabilis* BPS43, and four in *S. rubiginosohelvolus* NP10.

## 4. Discussion

Among different bacterial genera, *Streptomyces* are one of the most prolific producers of not only a variety of secondary metabolites with biomedical applications but also a variety of enzymes employed for biotechnological purposes, such as depolymerization and hydrolysis [51]. Following various enzyme classes, members of the *Streptomyces* genus are represented as versatile producers of different enzymes (cellulases, chitinases, xylanases, cutinases, etc.) [51]. Considering this vast biosynthetic potential, examination of *Streptomyces* degradation abilities on hard-to-deteriorate materials could prove very fruitful.

In this work, a degradation study of synthetic and natural textile materials was done, following the augmentation of model compost with three *Streptomyces* strains, based on prior screening. Compost has been chosen as the testing environment, since composting has been revealed to be an efficient and eco-friendly way of degrading different plastics [52], as well as varied textiles [53,54]. Taking into account weight loss results of PA material buried in augmented model compost, a small difference in mass during the degradation is in correlation with other studies referring to variation in degradation characteristics between polyamide-based materials, specifically PA4 and PA6 [55]. Unlike PA4, PA6 appeared more resistant in different soil and compost environments, with small changes in physical mass observed, but a more prominent change in molecular weight occurred [55]. In the case of PU materials, the composition plays a significant role in affecting degradation ability, with poly(ester-urethanes) showing to be more vulnerable to microorganisms available in compost, compared to the poly(ether-urea-urethane) [56], which was in accordance with our results. Additionally, weight loss in cotton in nonaugmented composting conditions is in correlation with weight loss shown by Li et al. (~55–70% after 3 months of composting) [22]. However, the bioaugmentation with *Streptomyces* sp. NP10 exhibited higher weight loss in CO material for the same degradation time, compared to this [22], and other studies [21] where authors noted a weight loss of ~40% after 3 months. These results suggested that the substrate, CO in this case, was more prone to degradation in the presence of *Streptomyces* sp. NP10, compared to the other strains, dictates the efficiency of degradation. Moreover, weight loss results of both synthetic (PA and PA-EA) and natural (CO) materials are in correlation with the study done by Liu et al. showcasing that the type of polymer (textile material in our case) determines the microbial community in complex environments such as soil and compost, and affects their metabolic processes and degradation abilities [57].

Comparison of the chemical structures of virgin and degraded materials was done by FTIR analysis. Representative FTIR spectra of PA are characterized by several peaks, which are consistent with other studies [58]. The peak located at 1640 cm^−1^ corresponds to the stretching vibration of the carbonyl group (C=O) in the amide I band [59], while the characteristic amide II band (N-H bending in combination with C-N stretching) is detected at 1535 cm^−1^ [60,61]. Characteristic amide appearing at ~1240 cm^−1^ is associated with the amide III band (C-N stretching and N-H bending vibrations in the amide group). The broad peak in the area of 3300–3400 cm^−1^ was due to the N-H stretching vibration from the amide groups, and the region at approximately 2850–2950 cm^−1^ came from C-H stretching in the aliphatic chain of the polymer backbone (alkyl-CH_2_, -CH_3_) [59].

In addition, bending vibrations of the -CH_2_ and -CH_3_ groups from PA are also visible at around 1450 cm^−1^, as well as C-C stretching at 1100 cm^−1^ in the polymer backbone [60]. The additional peak at 1730 cm^−1^ is coming from the stretching vibration of the carbonyl (C=O) group in the urethane functional group (-NH-C=O) [62]. From the presented FTIR spectra of PA material degraded in augmented compost (with *Streptomyces* sp. BPS43), the structure of the polymer remained unchanged, which could be attributed to the small weight loss in the material (16 wt% after 12 months compared to the nonaugmented compost) and these small changes were probably below the sensitivity limit to be detected. Analyzing the spectra of PA-EA material degraded in augmented model compost, the intensity of the characteristic peaks ranging from 3000 to 2800 cm^−1^ has decreased, suggesting the changes in the structure, amide (-CONH) functional groups being the place of chemical hydrolysis within the polyamide chains (Figure 3a) [63].

Cotton, primarily being composed of cellulose (linked glucose units), has several specific peaks: the peak around 3300–3500 cm^−1^ corresponds to the stretching of -OH (hydroxyl group), while the peak at approximately 2800–3000 cm^−1^ is attributed to the -CH_2_ stretchings (alkyl group) from the cellulose backbone. The decrease of the peaks at 3308 cm^−1^ and 2892 cm^−1^ observed after the degradation is similar to the results in composting conditions found by Li et al. [22] The band located at approximately 1495 cm^−1^, corresponded to symmetric C–H bending [22], while the band located near 1309 cm^−1^ was from -CH_2_ vibrations in cellulose and hemicellulose [64]. Stretching of C–O–C groups was detected at a wavenumber of 1163 cm^−1^ [22,64] for cellulose, while the band at 1108 cm^−1^ corresponded to anti-symmetric in-plane stretching [65]. The C–O characteristic peak at approximately 1000–1100 cm^−1^ was attributed to the ether linkages between glucose units in the cellulose [64]. Finally, the region at approximately 600–1800 cm^−1^ was defined as a fingerprint region, with peaks unique to the structure of cellulose, and it can be used for the identification of the cotton material [66].

PA control samples (shown by FESEM images) are indicated by elongated, intertwined fibers of smooth surface [63], while visual changes of the PA fiber structure following the degradation in the augmented model compost show similarity to PA fiber degraded by a fungus, previously observed [67]. Comparing the morphology of virgin PA with that of PA-EA blend, two components are clearly distinguishable: the PA component exhibits a smooth, fibrous surface, while the EA component appears more rough [68]. After the degradation, changes observed for PA-EA are related to changes in PU material discovered by Liu et al. [57]. As for CO material, the smooth, well-defined fiber morphology of CO was completely disintegrated, and the degraded material showed a rougher surface with broken, damaged fibers, compared to the control sample. This pattern of CO degradation was in line with previous research [22,54]. Comparing the visual changes of CO material after 30 days of augmented degradation, with other studies, similar findings were reported, indicating remarkable destruction of the material, which was even more intensive after 60 days in model compost [69].

The luminescent bacteria assay using *Aliivibrio fischeri* is a standardized method for assessing ecotoxicity. It is primarily applied to evaluate the toxic potential of wastewaters, eluates, and leachates originating from waste deposits. This assay has also proven effective in determining the toxicity of pure compounds and chemical mixtures [70]. In this study, PA, PA-EA, and CO extracts were used to determine the toxicity of these textile materials in a test system using freeze-dried luminescent bacteria. The results indicated that the analyzed textile samples exhibited no detectable environmental hazard and did not pose an ecological threat. On the other hand, polyamide 6,6, formed through the condensation of hexamethylenediamine (HMDA) and adipic acid, may be biologically degraded into its original monomeric constituents. HMDA does not exhibit environmental persistence or bioaccumulative potential. While its toxicity towards fish and aquatic invertebrates is relatively low, the compound demonstrates pronounced toxic effects on algal species at a concentration of 15 mg/L [71]. Acute exposure studies indicate that adipic acid exerts moderate toxic effects on aquatic fauna, including fish and invertebrates, with a lethal dose observed at concentrations above 200 mg/L [72]. In our previous study, eight synthetic model compounds, representing intermediates of PU hydrolysis, were synthesized and evaluated for their toxicity and potential as substrates for novel biocatalyst screening. Two hydrolysis-derived model compounds exhibited moderate aquatic toxicity, with EC_50_ values of 53 µg mL^−1^ and 45 µg mL^−1^ against *Aliivibrio fischeri* [30].

While PA 6,6 is widely recognized as biocompatible and noncytotoxic in its solid form, potential risks may arise from releasing leachable substances and nanoparticles, particularly when incorporated into composite materials. In this study, a significant reduction (50%) in cell viability is observed upon exposure to 100% PA extract until the 50% extract of the PA sample ensured cell growth above 77%. Although specific data on PA 6,6 cytotoxicity in HaCaT cells are limited, keratinocyte-based models have been widely used to assess the irritant and cytotoxic potential of various compounds, including polymers [73].

Modifying and degrading PA and PU polymers (considered nonbiodegradable) has been proven possible by different hydrolytic enzymes [63]. The most prominent enzymes are esterases (EC 3.1) and hydrolases effective on C-N bonds (EC 3.5), with even highly specific enzymes such as urethanases (EC 3.5.1.75) and nylonases (EC 3.5.1.117) described for the degradation of these polymers [45]. Accordingly, these types of enzymes were searched in the genomes of the selected strains. Other soil strains proved capable of degrading synthetic polymers such as PA4 in environments such as compost or campus soil, thus proving the presence of different enzymes capable of degrading PA in soil bacteria strains [55]. In the case of PU degradation, an interesting finding documented by de Witt et al. 2023 [74]. identifies a mesophilic *Halopseudomonas formosensis*, capable of rapid degradation of Impranil DLN^®^-SD, at temperatures of up to 50 °C. Additionaly, from several hydrolases identified from the strain, one polyester hydrolase was shown to degrade Impranil DLN^®^-SD, and thus this enzyme was purified and characterized in detail.

Analysing the phylogenetic tree, within the esterase branch, two esterases were identified as homologs of the well-characterized PU-degrading enzyme, PudA from *Commamonas acidovorans*, showing 36% and 39% identity for *S. spectabilis* BPS43_Est and *Streptomyces* sp. R1_Est, respectively. Additionally, two PETases were clustered with the esterases, both displaying approximately ~70% identity with the Tcur_1287 PETase, previously reported to degrade PU [75]. Despite their high sequence identity, these enzymes likely do not play a significant role in situ PA/PU degradation, as Tcur_1287 has been documented to achieve less than 1% weight loss with purified enzymes [75]. The protease branch contained homologs of the nylonase NylC5, reported for efficient PA hydrolysis [76]. Each strain harbored a single homolog with sequence identities ranging between 40 and 48% suggesting these can conserve proteases between the three strains. Finally, the amidase branch contained the most enzymes, most of which were homologous to the polyamidase from *Nocardia farcinica*, with sequence identities ranging between 36% and 46% [77,78]. *S. spectabilis* BPS43 contributed the most with 8 homologs, more than the other two strains combined, indicating these enzymes were responsible for the degradation activity observed in composting experiments. Five of BPS43’s amidases were clustered with different amidases from both *S. rubiginosohelvolus* NP10 and *Streptomyces* sp. R1. However, three (BPS43_Amd3, BPS43_Amd4, and BPS43_Amd8) of the enzymes were distinct and seemed to be unique to the strain. Notably, BPS43_Amd8 was the only homolog of the metagenome-derived polyurethanases, UMG-SP-1 and UMG-SP, which have been identified as some of the most active PU-degrading enzymes to date [79] with 44% identity. Given that BPS43_Amd8 is unique to *S. spectabilis* BPS43 and its similarity to benchmark polyurethanases, this enzyme is likely a key contributor to the PA/PU degradation activity observed.

Cellulose degradation by bacteria is performed by carbohydrate-active enzymes (CAZymes), mainly various glycosyl hydrolases [80]. They are responsible for the hydrolysis of glycosidic bonds between sugar monomers, or a sugar monomer and a nonsugar constituent [81]. *Streptomyces* are known as producers of various metabolites as well as CAZymes capable of degrading lignocellulosic substrates [51]. Although a wide range of CAZymes are common in *Streptomyces*, only several enzyme families are involved in cellulose degradation. While *Streptomyces* sp. R1 and *S. rubiginosohelvolus* NP10 both had 2 GH6 family members with CBM2, and GH6 enzymes in *S. spectabilis* BPS43 were without binding modules.

Interestingly, *Streptomyces* sp. R1 and *S. rubiginosohelvolus* NP10 both share a multimodular enzyme comprised of several catalytic domains capable of cellulose breakdown (GH5, GH6, and GH12), as well as a few cellulose-binding modules (CBM2, CBM3, CBM5, and CBM10). Despite *S. rubiginosohelvolus* NP10 not having the highest number of extracellular cellulases, this strain proved to be most effective in cotton degradation, in model compost burial tests (Figure 2). This result could indicate that *S. rubiginosohelvolus* NP10 is more adaptable to the specific characteristics and composition of model compost, and with more efficient CAZymes, where environmental factors were previously shown to be an important factor in CAZymes competence [50].

Our results identified *S. spectabilis* BPS43 as a potential degradation enhancer of PA and PA-EA textiles in composting conditions, with *S. rubiginosohelvolus* NP10 being effective in enhanced CO degradation. Additionally, genome mining showed the presence of several homologs of known amidases, as well as enzymes capable of degrading polyurethanes in *S. spectabilis* BPS43. Thus, using specific soil bacteria as biodegradation enhancers of synthetic textiles in environments such as soil can serve as a theoretical implication of our study. On the other hand, the identification of several enzymes homologous to known degraders of polyamides and polyurethanes could be the base for future research of identifing potent enzymes, optimizing their activity in natural environments, and designing efficient biocatalytic agents, capable of being used on a larger scale.

## 5. Conclusions

This research focused on the biodegradation potential of three *Streptomyces* strains, on PA, PA-EA, and CO, in burial tests in model compost. Weight loss results indicate *S. spectabilis* BPS43 as a promising strain with the ability to increase the degradation of PA, and PA-EA (16 and 10%, respectively), while S. *rubiginosohelvolus* NP10 accelerated the biodegradation of cotton textile by 14%. The genomic insights of the hydrolyzing machinery of the three *Streptomyces* strains revealed the presence of putative enzymes that can be further characterized and optimized, out of which the *S. spectabilis* BPS43 had the highest number of PA/PU degrading homologs (14), with eight enzymes found to be homologous to the polyamidase from *Nocardia farcinica*.

Furthermore, while the *S. rubiginosohelvolus* NP10 genome did not reveal the highest number of extracellular cellulases, this strain proved to be most effective in CO degradation during the burial tests warranting further assessment of its enzymes. Taken together, our results show the developing perspective of *Streptomyces* members in aiding the degradation of both synthetic and natural textiles, and thus proving them to be valuable tools that could be further optimized as whole-cell biocatalysts, or possibly integrated into the materials to be activated upon disposal.

## Data Availability

The raw data supporting the conclusions of this article will be made available by the authors on request.

## Supplementary Materials

The following supporting information can be downloaded at: https://www.mdpi.com/article/10.3390/microorganisms13081800/s1. Figure S1: Experimental setup of biodegradation under composting conditions: textile materials (PA and PA-EA) before the degradation in model compost; Figure S2: Visual changes of degraded textile materials: (A) PA and (B) PA-EA after 12 months of incubation; in nonaugmented model compost (MC) and augmented with *Streptomyces* sp. R1, *Streptomyces* sp. BPS43, and *Streptomyces* sp. NP10 (R1, BPS43, and NP10); Figure S3: Visual changes of degraded CO textile materials after 60 days of incubation; in nonaugmented model compost (MC) and augmented with *Streptomyces* sp. R1, *Streptomyces* sp. BPS43, and *Streptomyces* sp. NP10 (R1, BPS43, and NP10); Figure S4: Microbial growth in model compost, at the beginning of the soil burial test (Initial CFU), and after 12 months of degradation, for nonaugmented and model compost augmented with *Streptomyces* sp. BPS43, presented as logarithmic values; Figure S5: Photographs of CFU grown on three type of culture media (LA for heterotrophic bacteria; MSF for sporulating bacteria; SAB for fungi), for samples of nonaugmented model compost before the degradation (0 days), and nonaugmented model compost, as well as model compost augmented with *Streptomyces* sp. BPS43, after the degradation (12 months); Figure S6: Overlapped chromatograms of control and BPS43-treated samples, filtered for masses 227.3 and 453.3 *m*/*z*. (a) PA sample; (b) PA-EA sample; Figure S7: KEGG pathways annotation for selected strains: (a) *Streptomyces* sp. R1; (b) *Streptomyces spectabilis* BPS43; (c) *Streptomyces rubiginosohelvolus* NP10; Table S1: Masses of known PA and PU degradation products; Table S2: Overview of relevant genomic characteristics of selected strains; Table S3: Summary of hydrolytic enzymes relevant for PA and PU degradation in the genomes of selected strains; Table S4: Best hits of Blastp search for known PA/PU degrading homologs in the genomes of R1, BPS43 and NP10; Table S5: Analysis of total carbohydrate-active enzymes (CAZymes) found by dbCAN and filtered into those found by all three tools (HMMER, dbCAN_sub, and DIAMOND), with enzyme commission (EC) number, with signal peptides, and with the ability of using cellulose as substrate.
