# Supplementary material for: Degradation of Synthetic and Natural Textile Materials Using Streptomyces Strains: Model Compost and Genome Exploration for Potential Plastic-Degrading Enzymes
Source: Microorganisms. 2025 Aug 1;13(8):1800. doi: 10.3390/microorganisms13081800 (PMC12388512; doi:10.3390/microorganisms13081800)
Supplement: Supplementary file 1 [file microorganisms-13-01800-s001.zip › microorganisms-3714503-supplementary.pdf]

Supplementary material

Degradation of synthetic and natural textile materials using *Streptomyces* strains: Model compost and genome exploration for potential plastic-degrading enzymes

Vukašin Janković [1], Brana Pantelic [1], Marijana Ponjavic [1], Darka Marković [2], Maja Radetić [3], Jasmina Nikodinovic-Runic [1], Tatjana Ilic-Tomic [1,*]

1 Institute of Molecular Genetics and Genetic Engineering, University of Belgrade, Vojvode Stepe 444a, 11042 Belgrade, Serbia; vukasin.jankovic@imgge.bg.ac.rs (V.J.); brana.pantelic@imgge.bg.ac.rs (B.P.); marijana.ponjavic@imgge.bg.ac.rs (M.P.); jasmina.nikodinovic@imgge.bg.ac.rs (J.N.-R.)

2 Vinča Institute of Nuclear Sciences, National Institute of the Republic of Serbia, University of Belgrade, Mike Petrovića Alasa 12-14, Vinča, 11351 Belgrade, Serbia; darka.markovic@vin.bg.ac.rs

3 Faculty of Technology and Metallurgy, University of Belgrade, Karnegijeva 4, 11000 Belgrade, Serbia; ma-ja@tmf.bg.ac.rs

* Correspondence: tatjana.ilic-tomic@imgge.bg.ac.rs

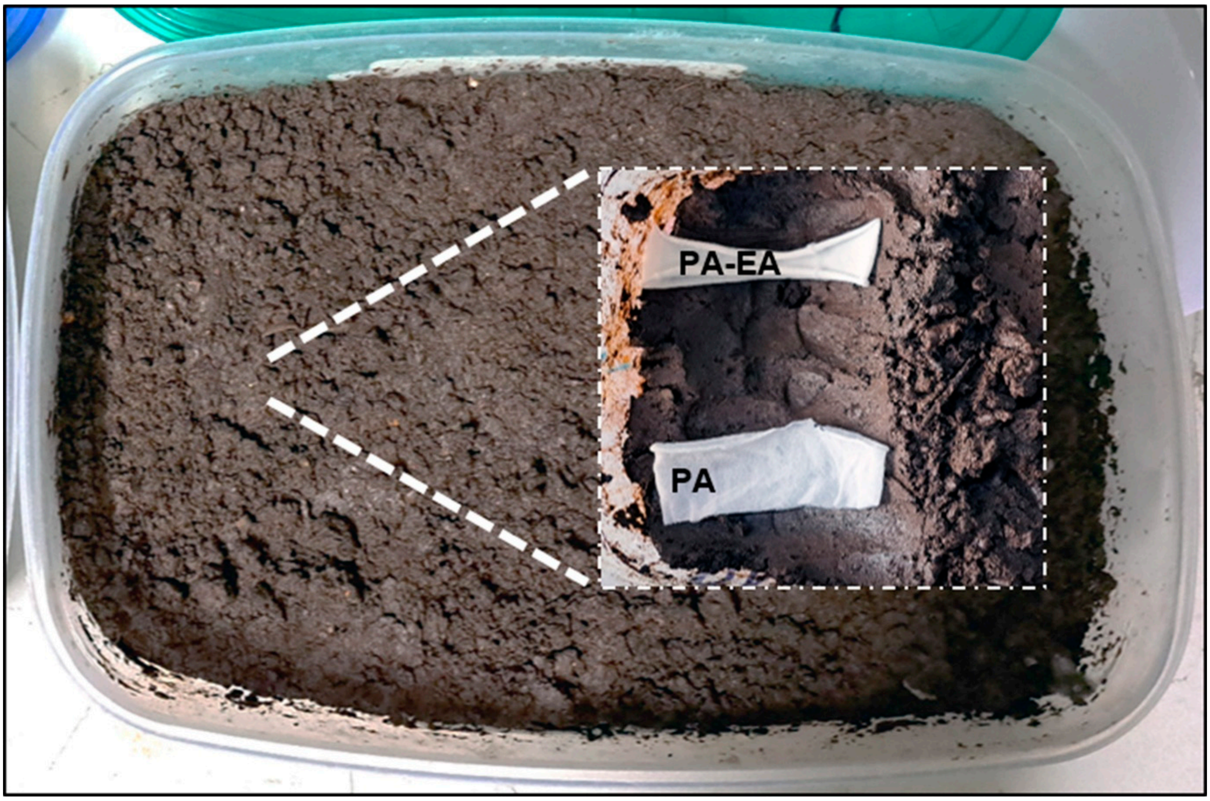

Figure S1. Experimental setup of biodegradation under composting conditions: textile materials (PA, and PA-EA) before the degradation in model compost.

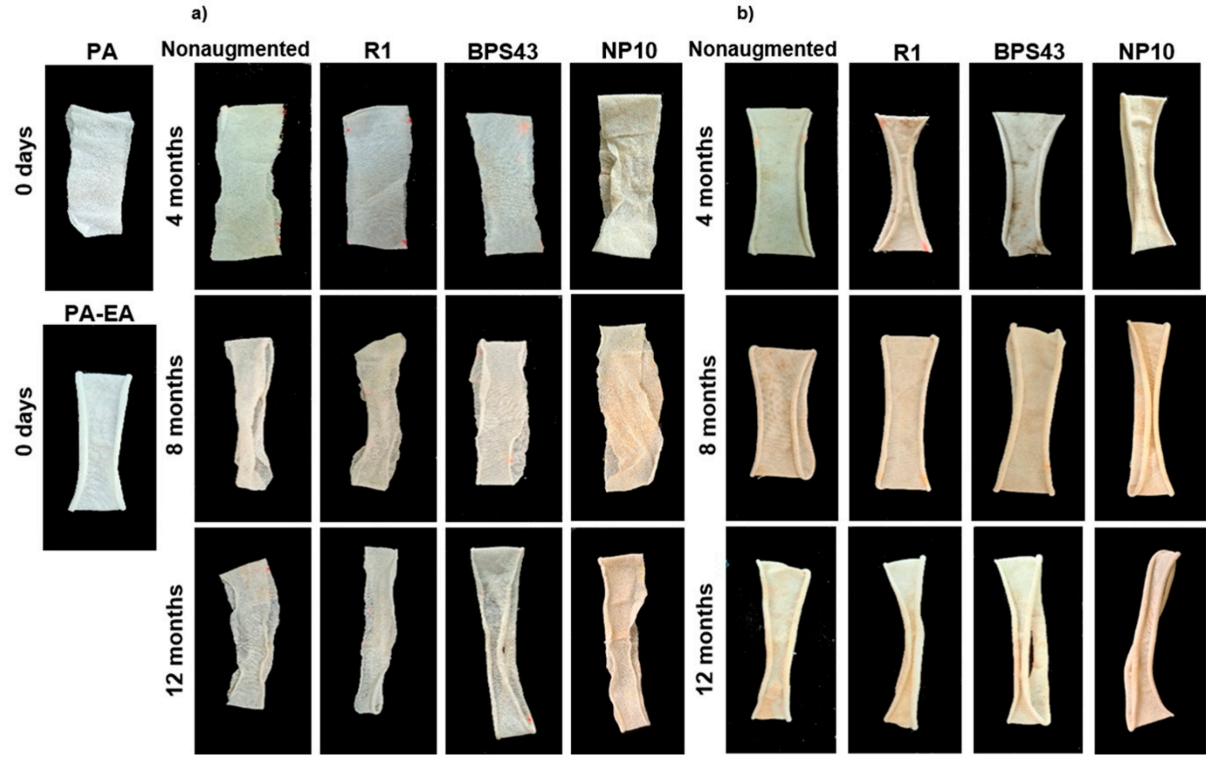

Figure S2. Visual changes of degraded textile materials: **A)** PA; **B)** PA-EA; after 12 months of incubation; in nonaugmented model compost (MC) and augmented with *Streptomyces* sp. R1, *Streptomyces* sp. BPS43, and *Streptomyces* sp. NP10 (R1, BPS43, and NP10)

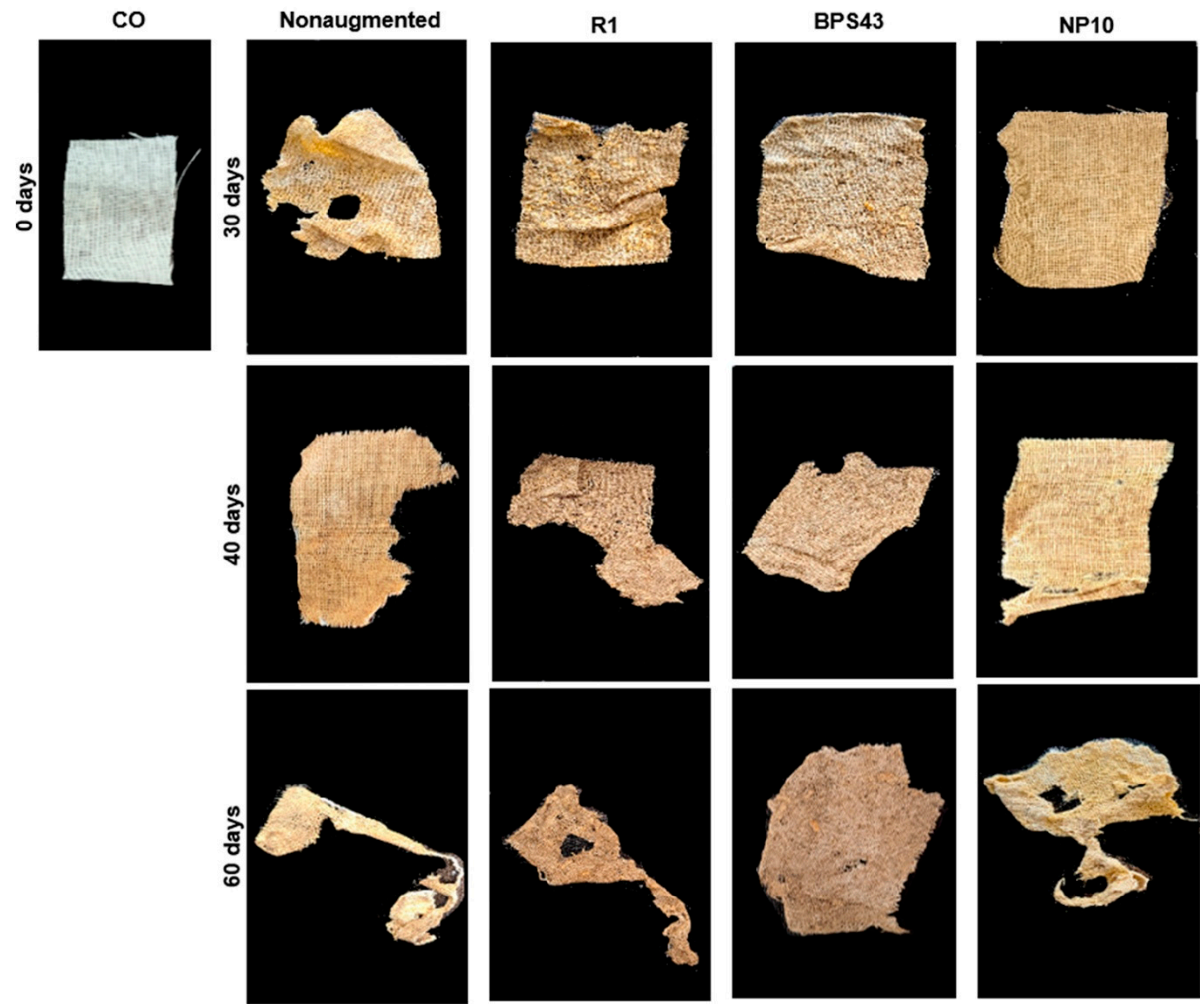

Figure S3. Visual changes of degraded CO textile materials after 60 days of incubation; in nonaugmented model compost (MC) and augmented with *Streptomyces* sp. R1, *Streptomyces* sp. BPS43, and *Streptomyces* sp. NP10 (R1, BPS43, and NP10)

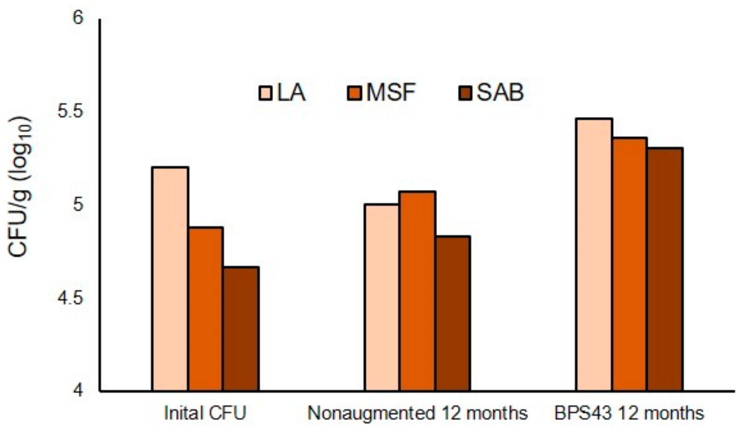

Figure S4. Microbial growth in model compost, at the beginning of the soil burial test (Initial CFU), and after 12 months of degradation, for nonaugmented and model compost augmented with *Streptomyces* sp. BPS43, presented as logarithmic values

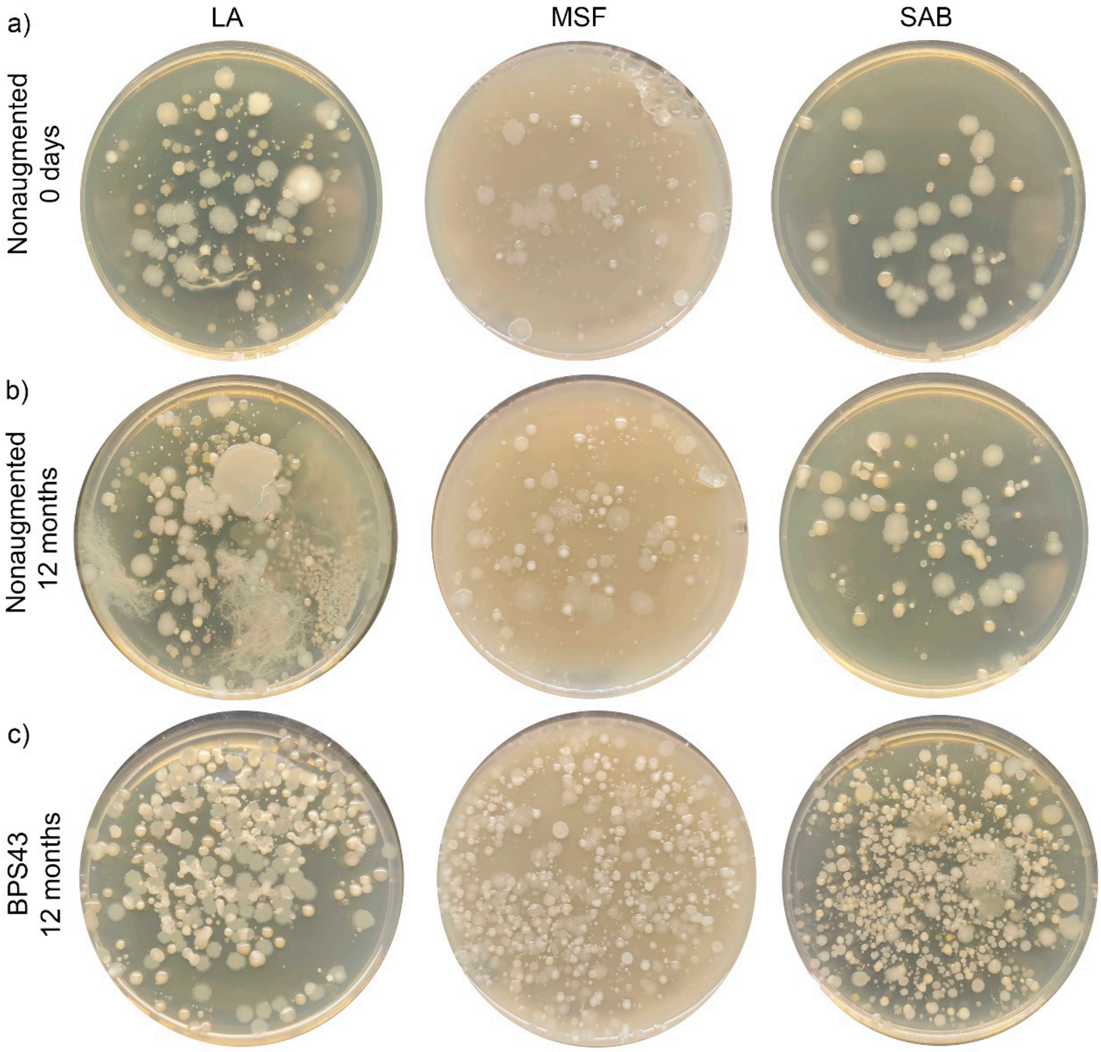

Figure S5. Photographs of CFU grown on three type of culture media (LA for heterotrophic bacteria; MSF for sporulating bacteria; SAB for fungi), for samples of nonaugmented model compost before the degradation (0 days), and nonaugmented model compost, as well as model compost augmented with *Streptomyces* sp. BPS43, after the degradation (12 months).

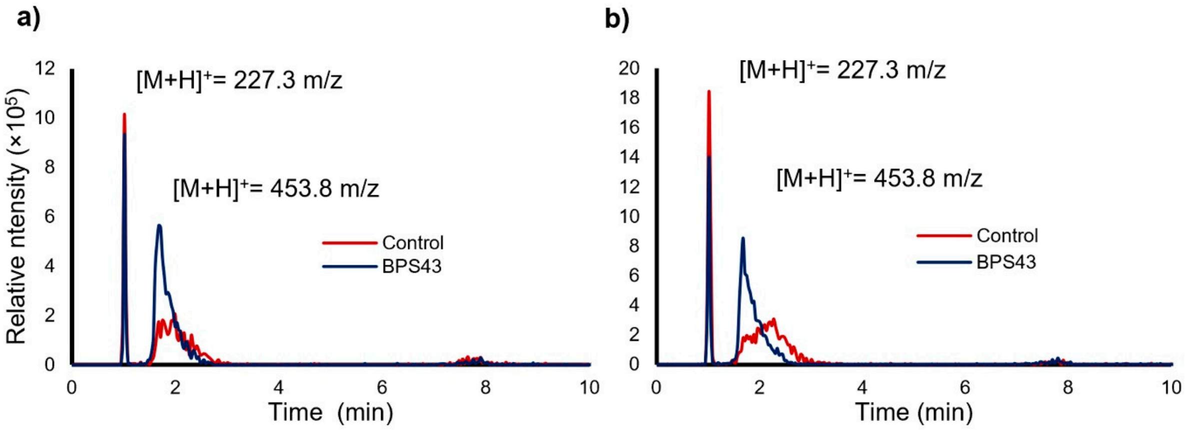

Figure S6. Overlapped chromatograms of control and BPS43-treated samples, filtered for masses 227.3 and 453.3 m/z. (a) PA sample; (b) PA-EA sample.

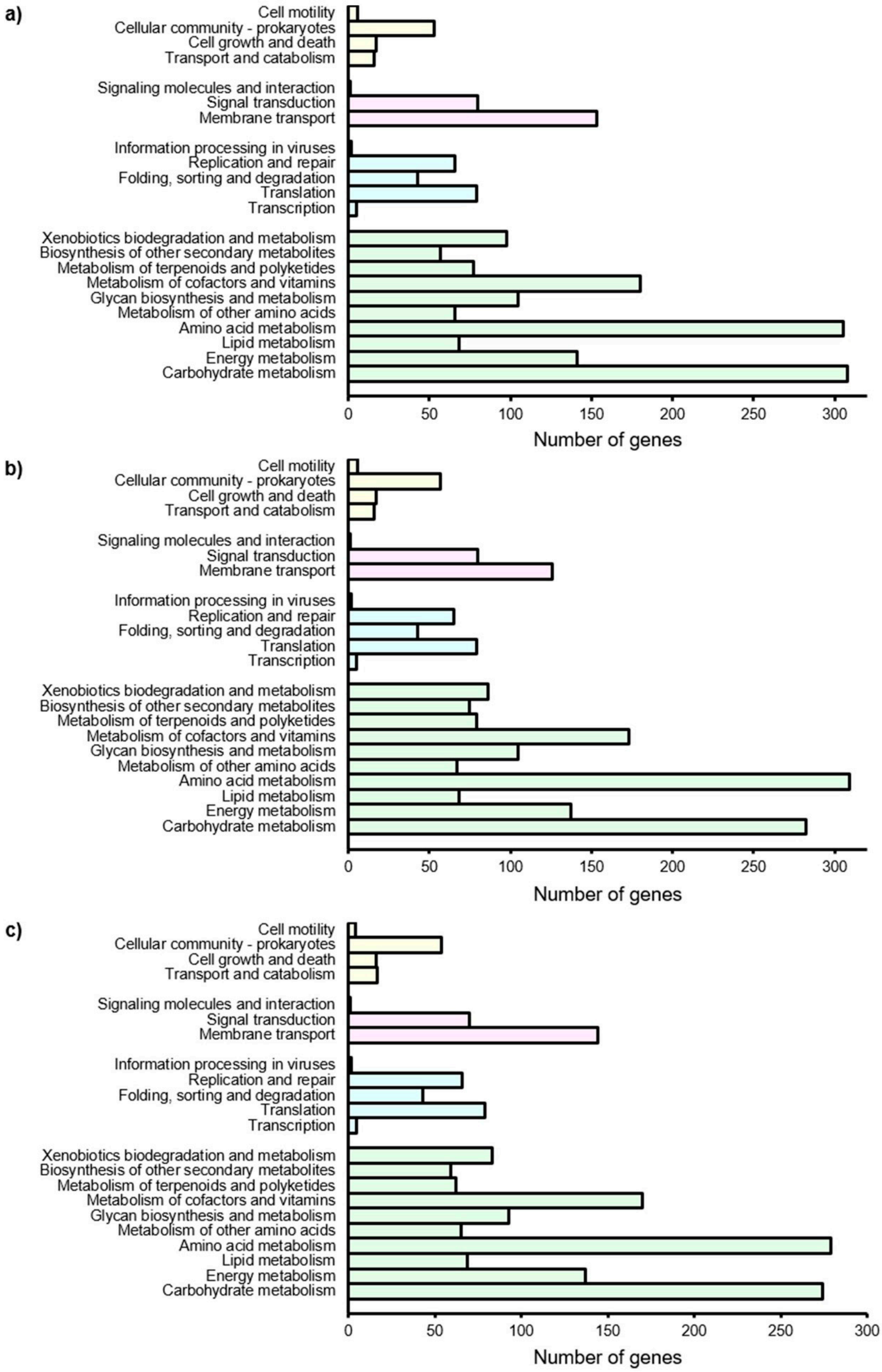

Figure S7. KEGG pathways annotation for selected strains: **a)** *Streptomyces* sp. R1; **b)** *Streptomyces spectabilis* BPS43; **c)** *Streptomyces rubiginosohelvolus* NP10

Table S1. Masses of known PA and PU degradation products

	Polymer degradation products	Molecular Weight
PA degradation products	PA6 dimer	227.3
	PA6 monomer (CPL)	113.6
	PA6 trimer	340.4
	PA6 tetramer	453.3
	PA6 pentamer	566.4
	PA6 hexamer	701.8
	PA66 monomer	227.3
	PA66 dimer	453.3
	PA66 trimer	701.8
PU degradation products	Adipic acid	146.14
	Diethylene glycol	106.12
	Ethylene glycol	62.7
	1,4-Butanediol	90.12
	Hexamethylene diamine	116.2
	4,4'-Methylenedianiline	198.26
	2,4-Diaminotoluene	22.17
	Neopentyl glycol	104.15
	6-hydroxyhexanoic acid	132.16
	Isophorone diamine	170.3
	Trimethylolpropane	134.17
	Propylene glycol	76.9

Table S2 - Overview of relevant genomic characteristics of selected strains

Metrics	***Streptomyces* sp. R1**	***S. spectabilis* BPS43**	**S. *rubiginosohelvolus* NP10**
**Accession number**	8122401480	8122886195	PDIQ01
**Size**	9.8 Mb	9.9 Mb	7.7 Mb
**Genes**	9120	8777	6934
**Protein coding**	8991	8605	6850
**GC content**	70.2%	72.4%	71.5%
**N50**	0.737 Mb	0.329 Mb	0.485 Mb
**Contig no.**	41	83	42
**Coverage**	84x	68x	73x
**Completeness**	99.50%	99.6%	99.9%
**Isolation environment**	*Cotinus coggygria* rizosphere	Vineyard soil	Woodland soil

Table S3 - Summary of hydrolytic enzymes relevant for PA and PU degradation in the genomes of selected strains.

Strain	EC. 3 (hydrolases)	EC. 3.1 (esterases)	EC. 3.5 (hydrolases acting on C-N bonds)
***Streptomyces* sp. R1**	458	111	102
***S. spectabilis* BPS43**	414	101	87
**S. *rubiginosohelvolus* NP10**	688	173	94

Table S4 - Best hits of blastp search for known PA/PU degrading homologs in the genomes of R1, BPS43 and NP10.

Strain	Enzyme (Accession no.)	Best hit	Accession number	Organism	Query cover (%)	Identity (%)	E-value	Active on
***Streptomyces* sp. R1**	R1_Est (8122402994)	PudA	BAA76305.1	*Comamonas acidovorans*	93	39.223	1.12E-98	PU
	R1_Amd3 (8122405950)	Amidotransferase	ARF18137.1	*Sporosarcina ureae*	94	39.246	2.4E-109	PU
	R1_Amd1 (8122406176)	Polyamidase	IMA10152A	*Nocardia farcinica*	99	43.532	2.3E-105	PA, PU
	R1_Pep (8122406465)	NylC5	XHH12378.1	*Gordonia sp.*	96	45.968	2.21E-56	PA
	R1_PETase (8122407205)	Tcur_1278	D1A9G5	*Thermobifida curvata*	86	69.261	8.5E-135	PU
	R1_Amd2 (8122408693)	Polyamidase	IMA10152A	*Nocardia farcinica*	97	43.461	1.2E-109	PA, PU
	R1_Amd4 (8122410120)	GatA urethanase	ANG60415.1	*Lysinibacillus fusiformis*	97	36.382	2.16E-83	PU
***S. spectabilis* BPS43**	BPS43_Amd6 (8122886303)	GatA urethanase	ANG60415.1	*Lysinibacillus fusiformis*	97	36.495	1.99E-83	PU
	BPS43_Pep (8122887564)	NylC5	XHH12378.1	*Gordonia* sp.	94	43.514	3.61E-60	PA
	BPS43_Amd2 (8122889295)	Polyamidase	IMA10152A	*Nocardia farcinica*	98	40.157	1.02E-89	PA, PU
	BPS43_Amd3 (8122889804)	Polyamidase	IMA10152A	*Nocardia farcinica*	80	36.203	4.76E-47	PA, PU
	BPS43_Amd4 (8122890040)	Polyamidase	IMA10152A	*Nocardia farcinica*	99	46.996	9.4E-123	PA, PU

	BPS43_Amd5 (8122890147)	Polyamidase	IMA10152A	*Nocardia farcinica*	90	42.797	2.4E-101	PA, PU
	BPS43_Amd7 (8122891294)	GatA urethanase	ANG60415.1	*Lysinibacillus fusiformis*	93	41.978	2.3E-107	PU
	BPS43_Amd8 (8122892111)	UMG-SP1	8S7Z	*Metagenome*	96	44.017	2.66E-89	PU
	BPS43_Est (8122892115)	PudA	BAA76305.1	*Comamonas acidovorans*	89	36.75	1.78E-78	PU
	BPS43_Amd1 (8122892910)	NylA	PSES8	*Pseudomonas* sp. NK87	95	36.308	1.67E-43	PA
***Streptomyces* sp. NP10**	NP10_Pep (RUP65510.1)	NylC5	XHH12378.1	*Gordonia* sp.	90	48.468	1.55E-53	PA
***Streptomyces* sp. NP10**	NP10_Hyd (RUP66616.1)	γ-lactamase	ACY56506.1	*Microbacterium hydrocarbonoxydans*	99	81.295	2.9E-177	PU
***Streptomyces* sp. NP10**	NP10_Amd1 (RUP67275.1)	Polyamidase	IMA10152A	*Nocardia farcinica*	84	36.947	8.04E-56	PA, PU
***Streptomyces* sp. NP10**	NP10_PETase (RUP67917.1)	Tcur_1278	D1A9G5	*Thermobifida curvata*	81	70.428	1.2E-136	PU
***Streptomyces* sp. NP10**	NP10_Amd1 (RUP70139.1)	GatA urethanase	ANG60415.1	*Lysinibacillus fusiformis*	97	37.32	3.91E-85	PU

Table S5 - Analysis of total carbohydrate-active enzymes (CAZymes) found by dbCAN, and filtered: to those found by all three tools (HMMER, dbCAN_sub, DIAMOND); with enzyme commission (EC) number; with signal peptides; with the ability of using cellulose as substrate.

Strain	CAZymes	3 tool consensus	With EC. number	Signal Peptide	Cellulases (EC 3.2.1.4)	GH6 family
***Streptomyces* sp. R1**	502	272	114	56	6	5
***S. spectabilis* BPS43**	444	183	69	34	3	3
***S. rubiginosohelvolus* NP10**	355	182	74	35	4	4
